# Human Mortality

**Published:** 1859-08

**Authors:** 


### HUMAN MORTALITY.

Using round numbers, of the four hundred thousand persons who died in England during 1856, fifty thousand died of consumption, most of the victims being young women. Nearly another fifty thousand died equally, from inflammation of the lungs, and bronchitis. Thus in England, one person out of every four, dies of diseases of the lungs.

Another clear fifty thousand, one out of every eight, died of diseases of the brain and nerves.

One out of every sixteen, died of diseases of the digestive organs.

It thus appears that ailments of the lungs first; next of the brain and nerves: next of the stomach, are the great destroyers of the people in civilized life, in the most cultivated nation on the globe.

About the same results are observed in the United States. Another classification might be made, carrying with it very instructive and admonitory suggestions.

The diseases of the breathing organs arise from physical causes; those from the brain and nerves are of a mental character; while those from the stomach are merely animal. But whether these three great slayers of the race, the physical, the mental, the animal, are not controllable to a great extent, who can doubt?

